# Chimney-Graft as a Bail-Out Procedure for Endovascular Treatment of an Inflammatory Juxtarenal Abdominal Aortic Aneurysm

**DOI:** 10.1155/2015/531017

**Published:** 2015-05-12

**Authors:** Francesca Fratesi, Ashok Handa, Raman Uberoi, Ediri Sideso

**Affiliations:** Department of Vascular Surgery, Oxford University Hospitals NHS Trust, Oxford OX3 9DU, UK

## Abstract

Inflammatory and juxtarenal Abdominal Aortic Aneurysm (j-iAAA) represents a technical challenge for open repair (OR) due to the peculiar anatomy, extensive perianeurysmal fibrosis, and dense adhesion to the surrounding tissues. A 68-year-old man with an 11 cm asymptomatic j-iAAA was successfully treated with elective EVAR and chimney-graft (ch-EVAR) without postprocedural complications. Target vessel patency and normal renal function are present at 24-month follow-up. The treatment of j-iAAA can be technically challenging. ch-EVAR is a feasible and safe bail-out method for elective j-iAAA with challenging anatomy.

## 1. Introduction

Inflammatory Abdominal Aortic Aneurysm (iAAA) is characterized by a thickened aortic wall and perianeurysmal fibrosis [1] with significant adhesions to the surrounding structures [2]. iAAAs are usually symptomatic and tend to present at a younger age with a triad of back pain, weight loss, and low grade fever. Elevated inflammatory markers with positivity of antinuclear antibody and elevation of IgG-4 plasma levels may be present [3]. Open repair (OR) remains the “gold standard” for treatment of iAAAs and juxtarenal aneurysms (jAAA), although there is an increased morbidity and mortality rate, longer operating time, and higher need for transfusions [4–7]. Endovascular repair (EVAR) offers an alternative as it obviates the need for extensive surgical dissection [4–6]. Fenestrated EVAR (f-EVAR) devices are used in jAAA to overcome the insufficient neck length resulting in inadequate sealing of standard endografts [8, 9]. Chimney-graft technique EVAR (ch-EVAR) was described to preserve the visceral aortic branches, deploying a stent parallel to the aortic endograft allowing the sealing in a healthier aortic zone [10]. A recent review of the ch-EVAR showed promising results in terms of morbidity, mortality, and durability at 6 and 12 months follow-up [11].

We present a unique case of a juxtarenal and inflammatory AAA (j-iAAA) successfully treated with ch-EVAR.

## 2. Case Presentation

**Figure 1:**
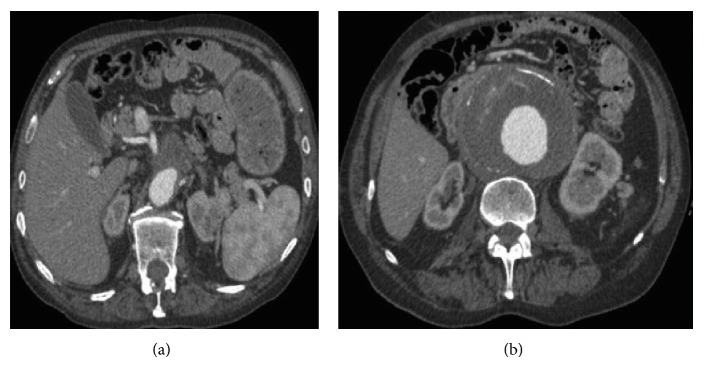
Preoperative CTA showing the extension of the AAA to the juxtarenal tract of the abdominal aorta (a) and the maximum diameter of the aneurysm (b). No signs of hydronephrosis were noted preoperatively.

**Figure 2:**
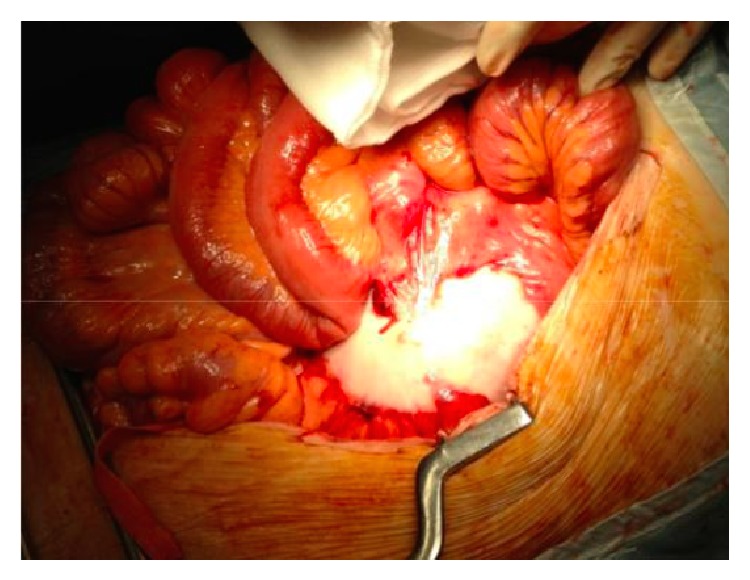
Intraoperative picture showing the thickened aortic wall and perianeurysmal fibrosis with significant adhesions to the surrounding structures encountered during the attempt of open repair.

**Figure 3:**
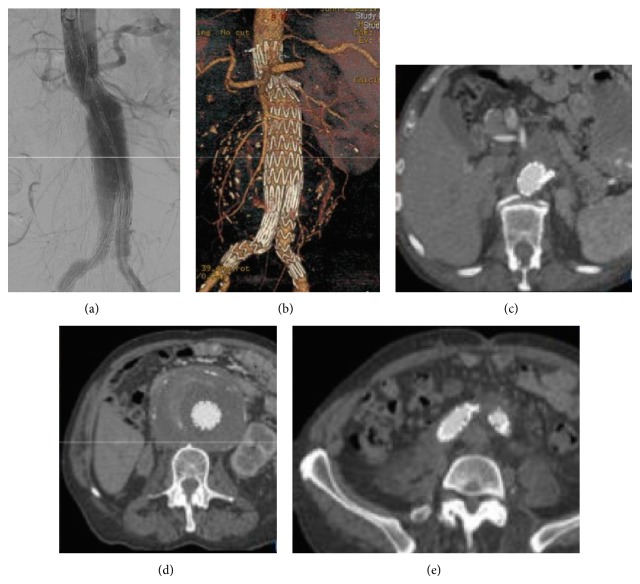
(a) Intraoperative completion angiography showing the good result of the ch-EVAR. (b) Reconstruction of postoperative CTA. Axial views of the postoperative CTA at the level of the origin of the left renal artery (c), mid-aneurysm (d), and bilateral common iliac arteries (e).

A 68-year-old man presented with several months' history of abdominal and back pain associated with a pulsatile abdominal mass. His comorbidity included hypertension, being a current smoker, and previous lung empyema. CT-angiography (CTA) showed an 11 cm j-iAAA with periaortic inflammation (PAI) involving the body and the neck of the AAA extending to the level of the origin of the superior mesenteric artery (SMA). The preoperative CTA did not show any signs of hydronephrosis associated with the retroperitoneal fibrosis (Figure 1). OR with longitudinal xifopubic access was proposed as treatment of choice, but the intraoperative findings revealed a dense fibrotic tissue surrounding the aorta making the dissection hazardous (Figure 2) and for this reason the OR was abandoned being deemed too high a risk for complications. Postoperatively, the patient had a prolonged recovery period due to recurrent lung empyema and respiratory complications but was discharged home on day 11. Considering the size of the j-iAAA and the risk of rupture still present, an endovascular solution was sought. The anatomy of the j-iAAA was deemed not suitable for standard EVAR considering the length of the neck (<1 cm) and its angulation (*α* angle > 60 degrees). A custom-made f-EVAR was considered but deemed unsuitable due to the right renal artery small size (<3 mm in diameter). A MAG3-Renogram demonstrated the dominant renal function of the left kidney (37% versus 63%) and guided the decision to sacrifice the small right renal artery. Always considering the size of the j-iAAA and the risk of rupture and the length of time necessary to have a custom-made graft with only one vessel fenestration, EVAR with single left renal artery chimney-graft (ch-EVAR) was considered as the preferable option. The ch-EVAR was performed under general anaesthesia, with bilateral percutaneous femoral approach and left brachial artery open access. The left renal covered-stent (Advanta V12, Atrium, 5 × 29 mm) chimney-graft was released following the deployment of the main body of the stent-graft (Zenith Flex, Cook, main body 30 × 140 mm, oversize 15%) below the SMA. A bifurcated stent-graft was then completed. The chimney-graft was reinforced with a bare metal stent (Protégè EverFlex, Ev3, 6 × 60 mm). Completion angiogram showed good position of the ch-EVAR with perfusion of the left kidney without any endoleaks. This was confirmed by CTA prior to discharge (Figure 3). Intraoperative blood loss was <500 mL. The patient was discharged on day 7 due to a recurrent lung empyema and need for a chest drain. No renal impairment was noted at the postoperative blood tests.

The follow-up was conducted with a Duplex Scan (DS) at 6 months which confirmed the patency of the ch-EVAR and the absence of endoleaks and the size of the j-iAAA was stable (11 cm). For this reason the follow-up CTA was conducted at one year, and it also confirmed the patency of the renal chimney-graft but it also revealed a late type 2 endoleak which was not present in the previous imaging. The CTA confirmed no aneurysm sac enlargement. The retroperitoneal periaortic inflammation (PAI) remained stable, without any signs of regression or progression noted at the CTA. The renal function was preserved at the blood test with also a maintenance of eGFR >90 mls/min/1.73 m^2^. At the time of publication of this case report the patient completed the 24-month follow-up: the CTA confirmed a stable type 2 endoleak without any signs of sac enlargement. Also the PAI was stable without any signs of renal complications or involvement. The renal chimney stent-graft is still patent and there are no signs of in-stent stenosis or extrinsic compression. The follow-up will be conducted yearly thereafter considering the stability of the AAA and the type 2 endoleak will be managed conservatively unless a complication such as sac enlargement or symptoms related to the AAA will appear during the follow-up.

## 3. Discussion

Juxtarenal and inflammatory aneurysms present challenging OR management and EVAR offers an alternative, with good short and mid-term results. ch-EVAR has been described as a bail-out option, particularly in urgent or emergent situations or when a standard or custom-made EVAR is not possible [7–10]. A recent review on ch-EVAR for jAAA reported an overall mortality of 3.4% at 30-day and 7.9% at 1-year follow-up [11]. The authors highlight that, at 6 months, the patency of the target vessels' chimney-grafts was 97.7%. Early type 1 endoleak was found in 7.4% of patients at completion of angiography and 10.2% at postoperative CTA; amongst this, 27.7% required treatment and 11.1% had a persistent type 1 endoleak [11]. Other authors reported a spontaneous regression of the leak in most cases (low-flow endoleak) at 12-month follow-up [12]. Late type 2 and type 3 endoleaks were present in 8.5% of the patients [11]. Good results of EVAR for iAAAs have been demonstrated in terms of short and mid-term morbidity and mortality, regression of PAI, and hydronephrosis [4, 12], but the benefit in the long term remains controversial. Paravastu et al. showed a trend for better outcome on mortality rate for EVAR compared to OR at 30 days (2% versus 6%, *p* = NS) and at 1 year (2% versus 14%, *p* = 0.01) [4]. Aneurysm related 1-year mortality was 0% for EVAR and 2% for OR (*p* = NS). In the subgroup of patients where hydronephrosis was analyzed, it was present in 48/85 (53%) of patients who underwent OR and 29/52 (56%) of patients who had EVAR; this regressed in 69% of OR and 38% of EVAR (*p* = 0.01), with progression observed in 9% and 21%, respectively (*p* = NS) [4]. At 1 year, PAI regressed in 73% of patients undergoing OR and 65% of patients treated with EVAR (*p* = 0.3) [4]. Stone et al. quantified the regression in their series and noted a mean decrease in the thickness of the inflammatory rind of 50.8% (range 0% to 92.1%) [5]. It has been hypothesised that the exclusion of the iAAA can help with regression of PAI. This is supported by the observed regression of PAI in 65% of patients treated with EVAR [4]. There is a suggestion that the endograft results in an inflammatory reaction in the aorta and this can be considerable over time although this normalizes after 12 months [13]. In a retrospective review of the EUROSTAR database, PAI was related to a higher incidence of graft thrombosis and limb stenosis (3.9% versus 0.3%, *p* = 0.00059) [7], explained by the thick fibrotic tissue making ballooning and modeling after deployment more difficult [6].

This case is unique as it presents a combination of two challenging issues for EVAR. Elective chimney-graft was used due to the adverse anatomical features of the AAA and failure at open repair.

This is the first published case in the literature of a ch-EVAR used as the primary treatment of a juxtarenal and inflammatory aneurysm. Long-term patency of the chimney-graft and resolution of PAI are ongoing concerns. Limb stenosis may also be a concern, given the high incidence of limb stenosis/occlusion reported in the EUROSTAR registry [6]. In this case PAI has not regressed to date, but patency of the target vessel chimney-graft remains. Considering the absence of hydronephrosis or ureteric insolvent at presentation of the AAA or during the follow-up and also considering the presence of history of recurrent lung empyema, the use of corticosteroid in this case was not considered for this patient. Also the management of choice for the type 2 endoleak was conservative as the size of the aneurysm remained stable at 2-year follow-up and the AAA is still asymptomatic.

## 4. Conclusions

Treatment of both inflammatory and juxtarenal AAAs can be technically challenging. Open repair remains the gold standard, but EVAR is feasible with good early and mid-term results. Elective ch-EVAR can be successfully used for the treatment of iAAA with challenging anatomy.
